# Variations of human heat shock proteins in multiple cancers

**DOI:** 10.1002/ctm2.320

**Published:** 2021-02-17

**Authors:** Xiaoxia Liu, Ka Li, Lingyan Wang, Miaomiao Zhang, Xiangdong Wang

**Affiliations:** ^1^ Zhongshan Hospital Institute of Clinical Science, Fudan University Shanghai Medical School; Jinshan Hospital Center for Tumor Diagnosis & Therapy, Jinshan Hospital, Fudan University Shanghai Medical School; Shanghai Engineering Research Center of AI Technology for Cardiopulmonary Diseases Shanghai Institute of Clinical Bioinformatics Shanghai China

Dear Editor,

The sensitivity of lung cancer cells to drugs was recently found to be associated with the expression of some heat shock proteins (HSP), of which the heat shock 70 kDa protein 6 (HSPA6) plays a decisive role in cell sensitivity through inter‐molecular communications among HSPAs, HSPs, and other signals.^1^ The major groups of HSPs, classified on basis of the molecular weights, are involved in the regulation of angiogenesis, cell proliferation, migration, invasion, and metastasis.^2^, ^3^ HSPs may promote cell toxicity and resistance to drugs^4^, ^5^ and can be used as cancer diagnostic and prognostic biomarkers, as well as therapeutic targets for clinical practice, to represent the specificity of disease, stage, duration, severity, and response to drugs.^6^, ^7^, ^8^ In order to furthermore clarify roles of HSPs in cancers, we comprehensively analyzed transcriptomic profiles of HSP family, e.g. chaperonins, DNAJ (HSP40), HSPA (HSP70), HSPC (HSP90), and HSPB (small HSP), in 9018 patients with 28 cancers.

To identify cancer‐related HSPs, differentially expressed genes (DEG) of cancer (n = 9018) and normal tissues (n = 5526) were assessed in 28 cancers with *P* < .05 and fold changes > 1.5 folds. The full frequency of 95 HSPs in databases was 2660 in 28 cancers, while about 46% of HSPs were up‐ and down‐regulated in each cancer. This implies that HSP family members actively or passively contribute to the development of cancer. Table 1 demonstrates that more than 40 HSPs were dysregulated in 16 cancers, including squamous cell carcinoma, lymphoma, and gastrointestinal adenocarcinoma. The results showed more than 70% cancer‐related HSPs were up‐regulated in thymoma (n = 75), diffuse large B‐cell lymphoma (DLBC) (n = 74), and pancreatic adenocarcinoma (n = 71), while also about 50% were down‐regulated in testicular germ cell tumors (TGCT) (n = 63), and acute myeloid leukemia (n = 60). It seems that dysregulated expression of HSPs may mainly contribute to leukocyte‐ and epithelium‐origin cancers. Figure 1A demonstrated that HSPA6 was highly down‐regulated in thymoma and DLBC, while over‐expressed in lung adenocarcinoma (LUAD). The deletion of HSPA6 made lung cancer cells resistant to the therapy (1). DNAJ sub‐family members were the major part of those down‐regulated HSPs. Up‐ or down‐regulation of DNAJ protein expression can have bi‐directional functions to promote or suppress cancer development by regulating cancer stem cell differentiations in many cancer tissues. DNAJ chaperonins band with cAMP‐dependent protein kinase catalytic subunit (Cα) to form a chimeric enzyme, to interact with HSP70 through the signals of the proto‐oncogene A‐kinase anchoring protein‐Lbc with a RAF‐MEK‐ERK kinase module.^9^ On average, there were about 44 DEG in each cancer, and each HSP was associated with about 13 cancers (Table 1, Supplemental Table 1). In total, 31 HSPs were differentially expressed in 17/28 cancer types (>60%), and 28 in 8 cancers (<30%) (Table S1).

**TABLE 1:** The number of patient tumors and corresponding normal tissues and differentially expressed genes (DEG) of dysregulated (sum of upregulated and downregulated), upregulated, or downregulated heat shock proteins (HSPs) with *P*‐values less than 0.05 and changed folds > 1.5, as compared with levels of normal tissues per cancer type, respectively

Cancers	Number of samples		Dysregulated		Upregulated		Downregulated	
	Tumor	Normal	DEG No.	Percent	DEG No.	Percent	DEG No.	Percent
THYM	118	339	75	79%	73	77%	2	2%
DLBC	47	337	74	78%	70	74%	4	4%
PAAD	179	171	71	75%	70	74%	1	1%
TGCT	137	165	63	66%	22	23%	41	43%
LAML	173	70	60	63%	12	13%	48	51%
READ	92	318	56	59%	38	40%	18	19%
COAD	275	349	54	57%	36	38%	18	19%
GBM	163	207	53	56%	43	45%	10	11%
BRCA	1085	291	48	51%	36	38%	12	13%
STAD	408	211	48	51%	43	45%	5	5%
ESCA	182	286	47	49%	36	38%	11	12%
SKCM	461	558	47	49%	38	40%	9	9%
UCEC	174	91	46	48%	22	23%	24	25%
UCS	57	78	44	46%	24	25%	20	21%
LUSC	486	338	43	45%	27	28%	16	17%
CESC	306	13	40	42%	24	25%	16	17%
LIHC	369	160	39	41%	36	38%	3	3%
OV	426	88	39	41%	15	16%	24	25%
LGG	518	207	38	40%	31	33%	7	7%
KICH	66	53	35	37%	9	9%	26	27%
PRAD	492	152	32	34%	16	17%	16	17%
BLCA	404	28	31	33%	14	15%	17	18%
HNSC	519	44	31	33%	26	27%	5	5%
LUAD	483	347	27	28%	11	12%	16	17%
KIRC	523	100	25	26%	11	12%	14	15%
ACC	77	128	23	24%	9	9%	14	15%
KIRP	286	60	20	21%	12	13%	8	8%
THCA	512	337	19	20%	1	1%	18	19%
Total	9018	5526	1228		805		423	

ACC: Adrenocortical carcinoma; BLCA: Bladder Urothelial Carcinoma; BRCA: Breast invasive carcinoma; CESC: Cervical squamous cell carcinoma and endocervical adenocarcinoma; COAD: Colon adenocarcinoma; DLBC: Diffuse Large B‐cell Lymphoma; ESCA: Esophageal carcinoma; GBM: Glioblastoma multiforme; HNSC: Head and Neck squamous cell carcinoma; KICH: Kidney Chromophobe; KIRC: Kidney renal clear cell carcinoma; KIRP: Kidney renal papillary cell carcinoma; LAML: Acute Myeloid Leukemia; LGG: Brain Lower Grade Glioma; LIHC: Liver hepatocellular carcinoma; LUAD: Lung adenocarcinoma; LUSC: Lung squamous cell carcinoma; OV: Ovarian serous cystadenocarcinoma; PAAD: Pancreatic adenocarcinoma; PRAD: Prostate adenocarcinoma; READ: Rectum adenocarcinoma; SKCM: Skin Cutaneous Melanoma; STAD: Stomach adenocarcinoma; TGCT: Testicular Germ Cell Tumors; THCA: Thyroid carcinoma; THYM: Thymoma; UCEC: Uterine Corpus Endometrial Carcinoma; UCS: Uterine Carcinosarcoma.

FIGURE 1Differentially expressed HSPs genes in normal and cancer tissues (A) were categorized as high (red), moderate (white), and low (blue) expression. Patient overall survival rates were correlated with HSPs expression in 28 cancers (B), where the high density of red color represents the poor prognosis and blue color represents the good prognosis. HSP gene mutation frequencies and types in 1761 lung adenocarcinoma samples were defined from cBioPortal database (C)

**Figure:**
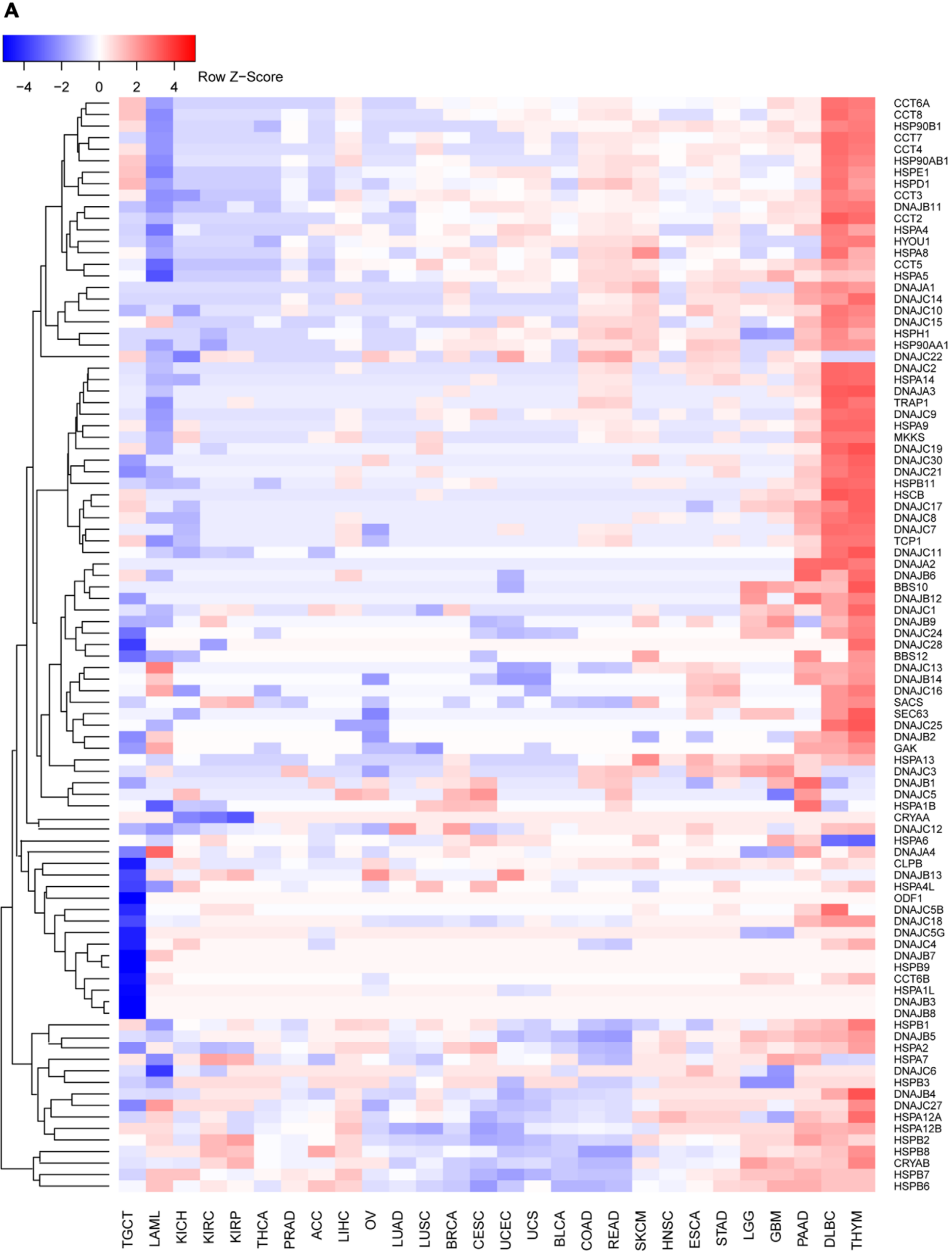

**Figure:**
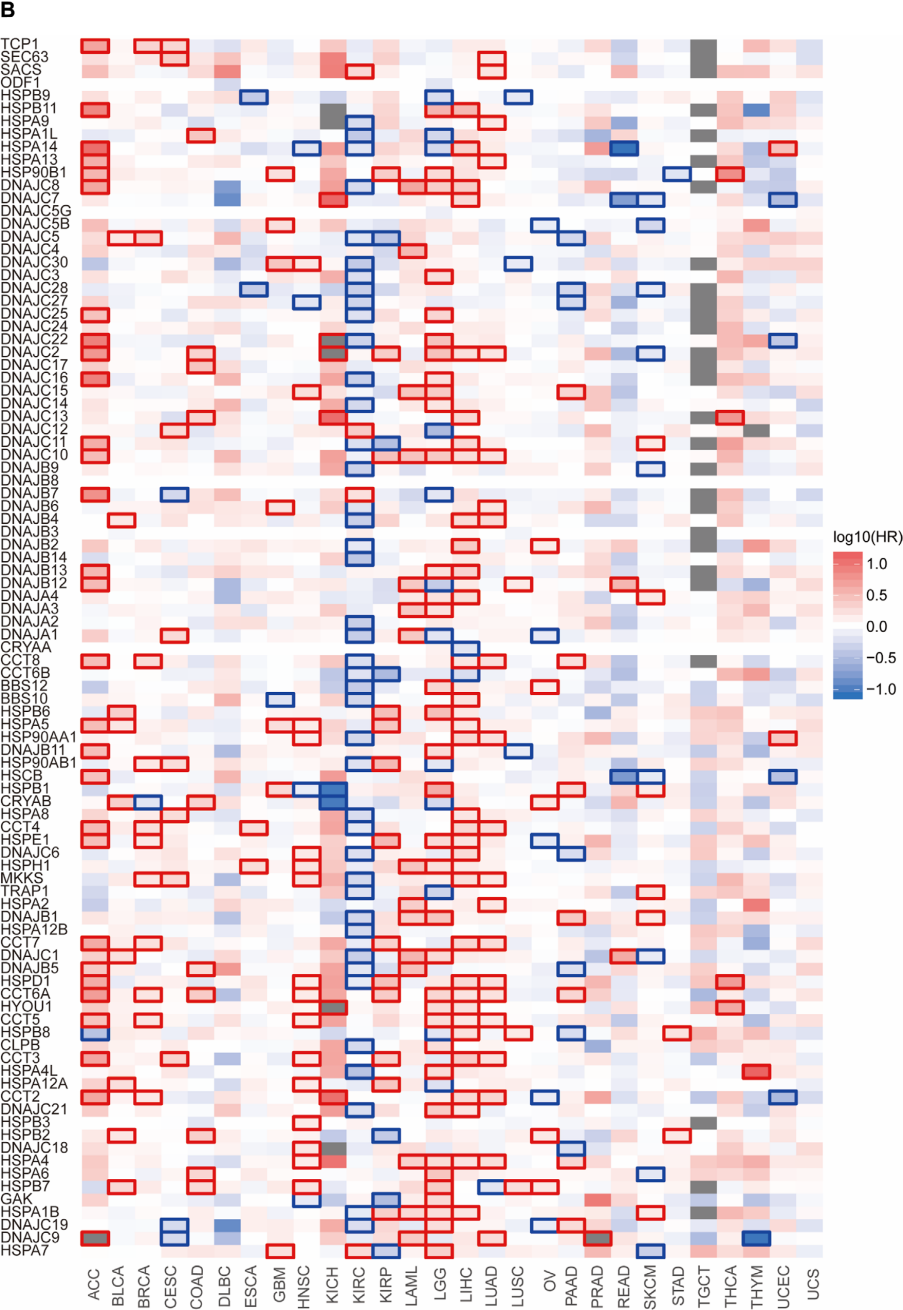

**Figure:**
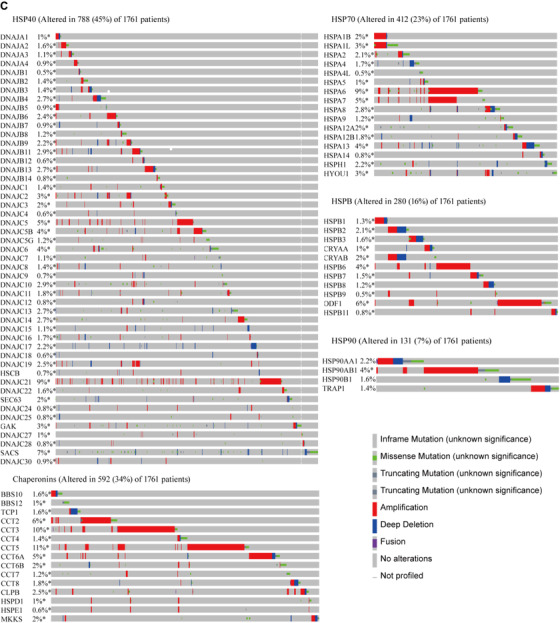

The HSPs associated with prognosis for cancer patients were identified using the Gene Expression Profiling Interactive Analysis (GEPIA2)^10^ database. The correlation between patient overall survival (OS) rate and HSPs expression was defined with the log‐rank test. The low‐expression and high‐expression of each HSP were categorized on basis of the median expression used as a cutoff. The highest number of survival‐associated HSPs was found in renal clear cell carcinoma (52 HSPs), whereas no HSP was significantly associated with prognosis for patients with DLBC or TGCT (Figure 1B). CCT6A, CCT7, and DNAJC9 had prognostic values in 8–9 different cancer types, rather than ODF1, DNAJB8, and DNAJC5G. In addition, we performed the HSPs mutation analysis in TCGA pan‐cancer and listed mutations of HSPs in Supplemental Table 2. To predict the possible mechanism of HSPs in cancer development, we summarized the pathways of HSPs (Figure 4).

Our previous study had found that the sensitivity of lung cancer cells to drugs was related to HSPs.^1^ LUAD is the most common subtype of lung cancer with high mortality and morbidity. Therefore, we selected LUAD to illustrate expression and function of HSP. We analyzed the expression of HSPs in 515 LUAD tissues and 59 control tissues from TCGA. Of those, 25 DEG‐HSPs were upregulated and 13 downregulated (*P* < .05, fold Changs > 1.5, Figure 2). To find the LUAD‐specific HSPs, we compared the expression of the 38 HSPs in LUAD with other 27 cancers and obtained four LUAD‐specific HSPs (HSPB1, CRYAB, HSPB2, DNAJC5B) (Table 2). In addition, other 25 upregulated HSPs and 13 downregulated HSPs had the diagnostic values to distinguish LUAD patients from controls (Figure 3A). Among upregulated genes, CCT3 had the highest value of area under curve (0.99), with the sensitivity and specificity of 94% and 98% (*P *< .0001), respectively. Among downregulated genes, HSPA12B was the highest (0.99), with the sensitivity and specificity of 94% and 98%, respectively (*P *< .0001; Table S3). To assess gene mutations of the HSP family in LUAD, 95 HSPs were examined in 1761 samples from 6 LUAD studies using the cBioPortal (https://www.cbioportal.org/). The mutation frequencies, types, and classifications of HSPs were shown in Figure 1C. Mutation frequencies of CCT5, CCT3, HSPA6, and DNAJC21 were significantly higher (11%, 10%, 9% and 9%, respectively). The frequency variation of HSPA4L, DNAJB1, and HSPB9 were the lowest (0.5%). Of 95 HSPs, 15 HSPs had significant correlations with OS rates in patients with LUAD (Figure 3B). High expression of HSPA2, DNAJC9, DNAJB4, HSPA4, CCT2, CCT4, CCT6A, DNAJC22, HSPD1, HSPA1L, TCP1, and DNAJC2 mRNA was associated with poor prognosis in LUAD patients. The high expression of DNAJB13 (HR 0.91, *P *= .04), HSPB7 (HR 0.90, *P *= .03), and DNAJC5 (HR 0.82, *P *= .003) were related to better long‐term prognosis (Figure 3C).

**FIGURE 2:**
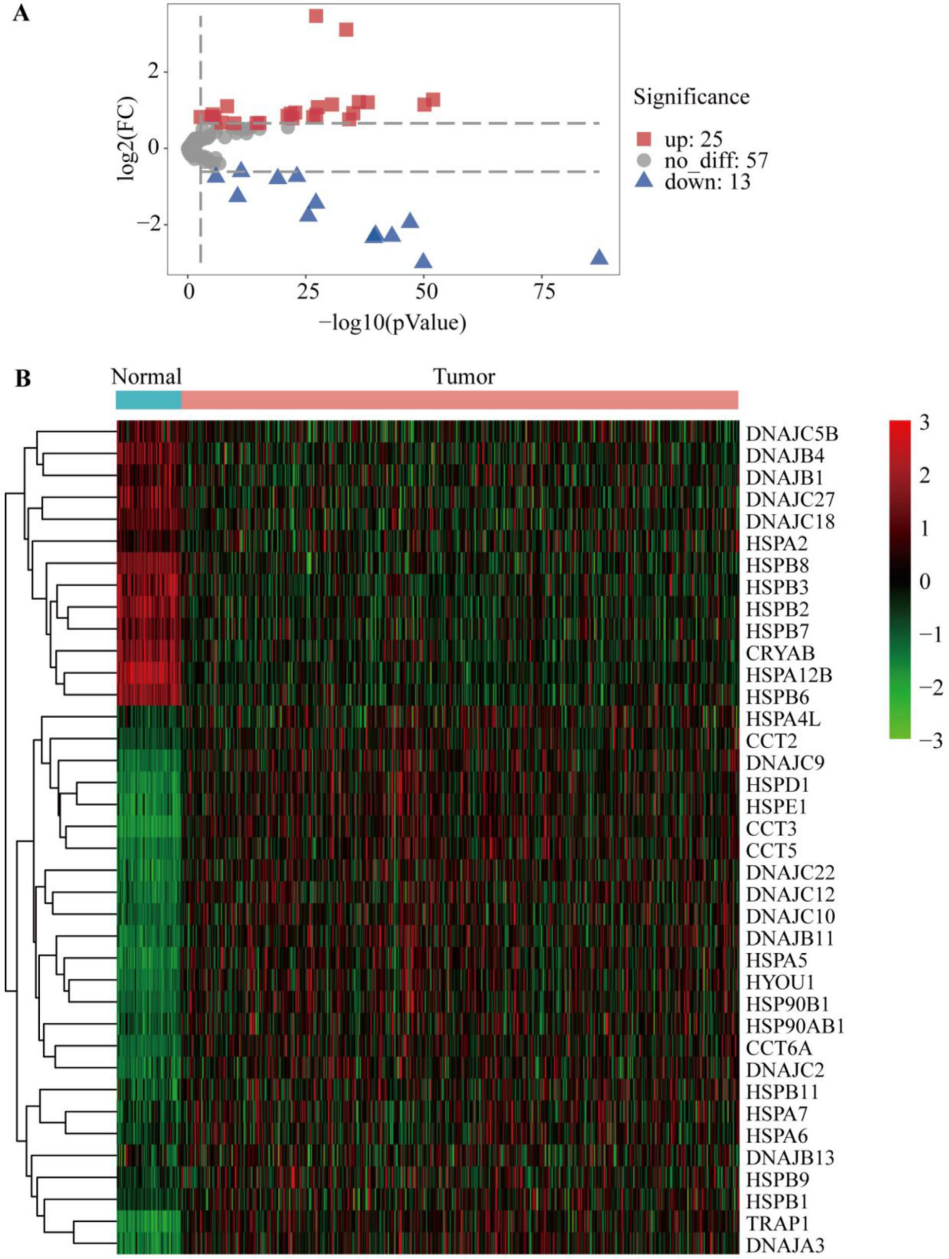
The differently expressed HSP genes in patients with LUAD from TCGA database. A, Volcano plot showing the differential expression of HSPs between the LUAD and normal tissues, including 25 up‐regulated and 13 down‐regulated HSPs. *X*‐axis: ‐log10 *P*‐value for each probe; *Y*‐axis: log_2_ fold change. B, Heatmap of the differential expression of validated HSPs in LUAD‐TCGA dataset. Red indicates high expression; black indicates moderate expression; green indicates low expression. Sample type: blue indicates normal controls; pink indicates LUAD patients

**TABLE 2:** The specificity of HSPs in LUAD compared with other 27 cancers

**Upregulated**				**Downregulated**			
Gene Symbol	logFC	P.Value	Cancer Number^#^	Gene Symbol	logFC	P.Value	Cancer Number^#^
DNAJC12	3.47	6.43E‐28	3	HSPB6	‐3.00	1.27E‐50	3
DNAJC22	3.11	2.63E‐34	2	HSPA12B	‐2.90	6.54E‐88	4
CCT3	1.28	1.05E‐52	4	HSPB3	‐2.34	3.96E‐40	1
HSPE1	1.21	5.59E‐37	4	CRYAB*	‐2.31	5.58E‐44	0
HSPD1	1.21	7.44E‐39	2	HSPB8	‐2.27	1.64E‐40	4
CCT5	1.15	2.84E‐31	3	HSPB2*	‐1.94	8.13E‐48	0
TRAP1	1.14	7.09E‐51	1	HSPB7	‐1.77	2.86E‐26	2
HSPA7	1.10	4.61E‐09	5	DNAJB4	‐1.43	7.06E‐28	3
HYOU1	1.08	2.75E‐28	4	DNAJC5B*	‐1.26	2.71E‐11	0
CCT6A	0.94	1.79E‐23	6	DNAJC18	‐0.79	8.42E‐20	3
HSPA5	0.92	8.60E‐36	5	HSPA2	‐0.75	1.08E‐06	2
HSP90B1	0.91	1.52E‐22	1	DNAJC27	‐0.74	7.40E‐24	4
HSPB9	0.89	5.37E‐06	8	DNAJB1	‐0.61	5.01E‐12	3
DNAJC9	0.88	6.19E‐28	5				
DNAJC10	0.86	7.41E‐22	3				
HSPA4L	0.83	8.36E‐06	5				
DNAJB13	0.82	1.89E‐03	2				
DNAJB11	0.82	2.85E‐27	4				
HSPA6	0.80	2.11E‐06	3				
DNAJC2	0.78	6.84E‐23	7				
DNAJA3	0.76	6.52E‐35	3				
HSPB1*	0.68	8.09E‐08	0				
HSP90AB1	0.67	6.81E‐16	3				
HSPB11	0.66	2.47E‐15	7				
CCT2	0.66	1.30E‐10	5				

^#^ Number of cancers with no statistically significant difference in gene expression compared to LUAD.

* LUAD‐specific expressed HSP.

FIGURE 3Validation of clinical significance of HSPs in LUAD‐TCGA patients. A, Receiver operating characteristic (ROC) curves analysis of HSPs for the diagnosis of LUAD. *X*‐axis: 100‐specificity%; *Y*‐axis: sensitivity%. B, Overall survival analysis of HSPs in LUAD patients was performed using the Kaplan–Meier method and log‐rank test. The median value of HSPs expression was defined as cut‐off value between high expression and low expression. C, Overall forest plots of differentially expressed HSPs in patients with LUAD

**Figure:**
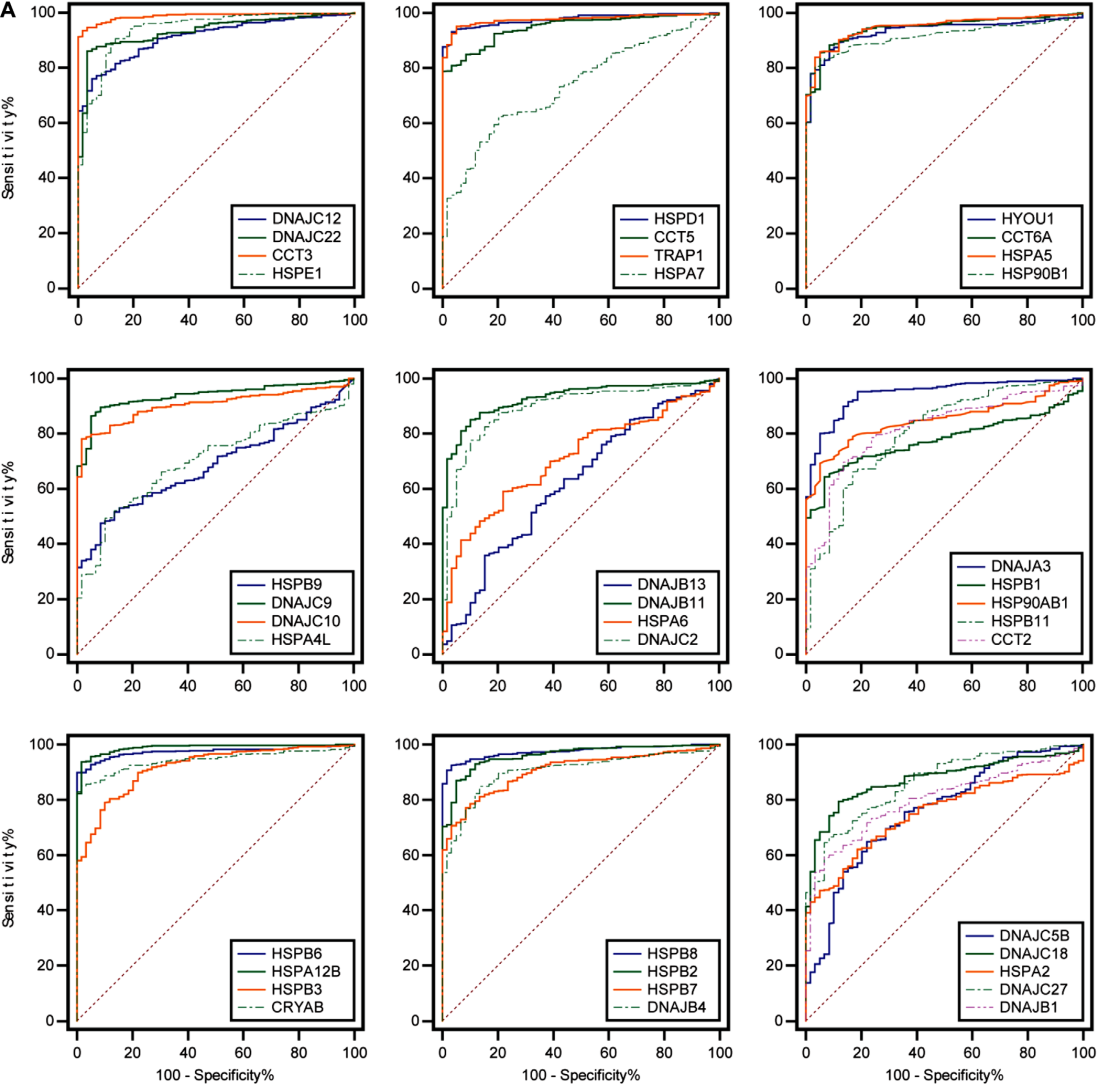

**Figure:**
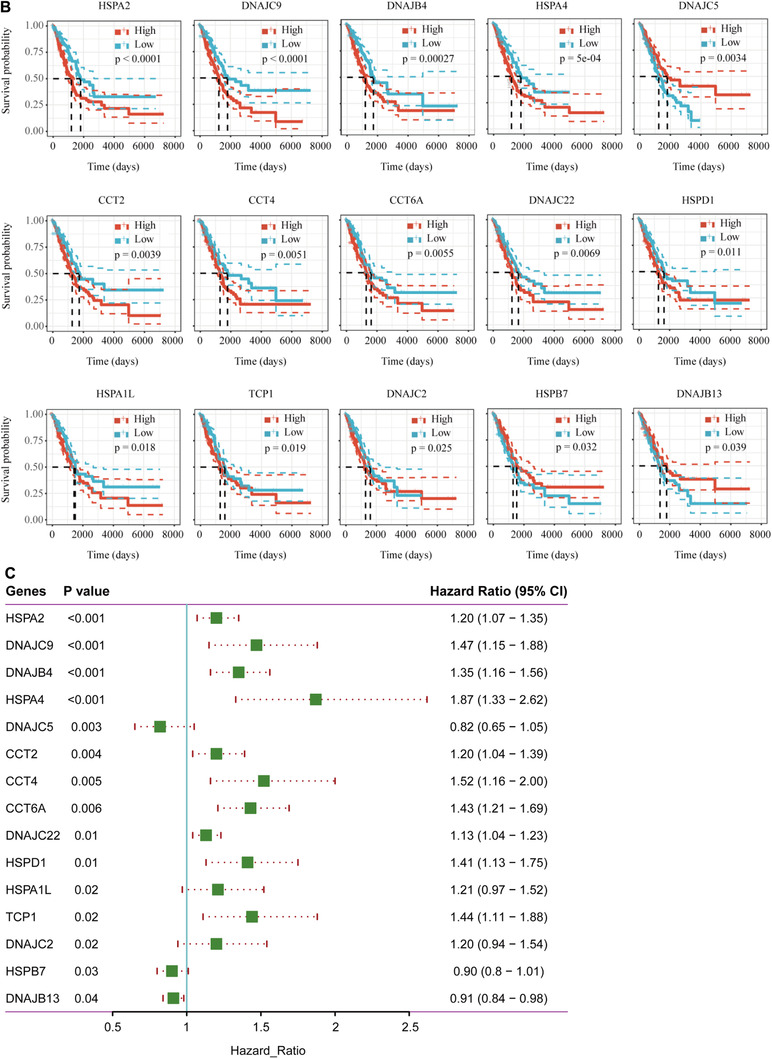

**FIGURE 4:**
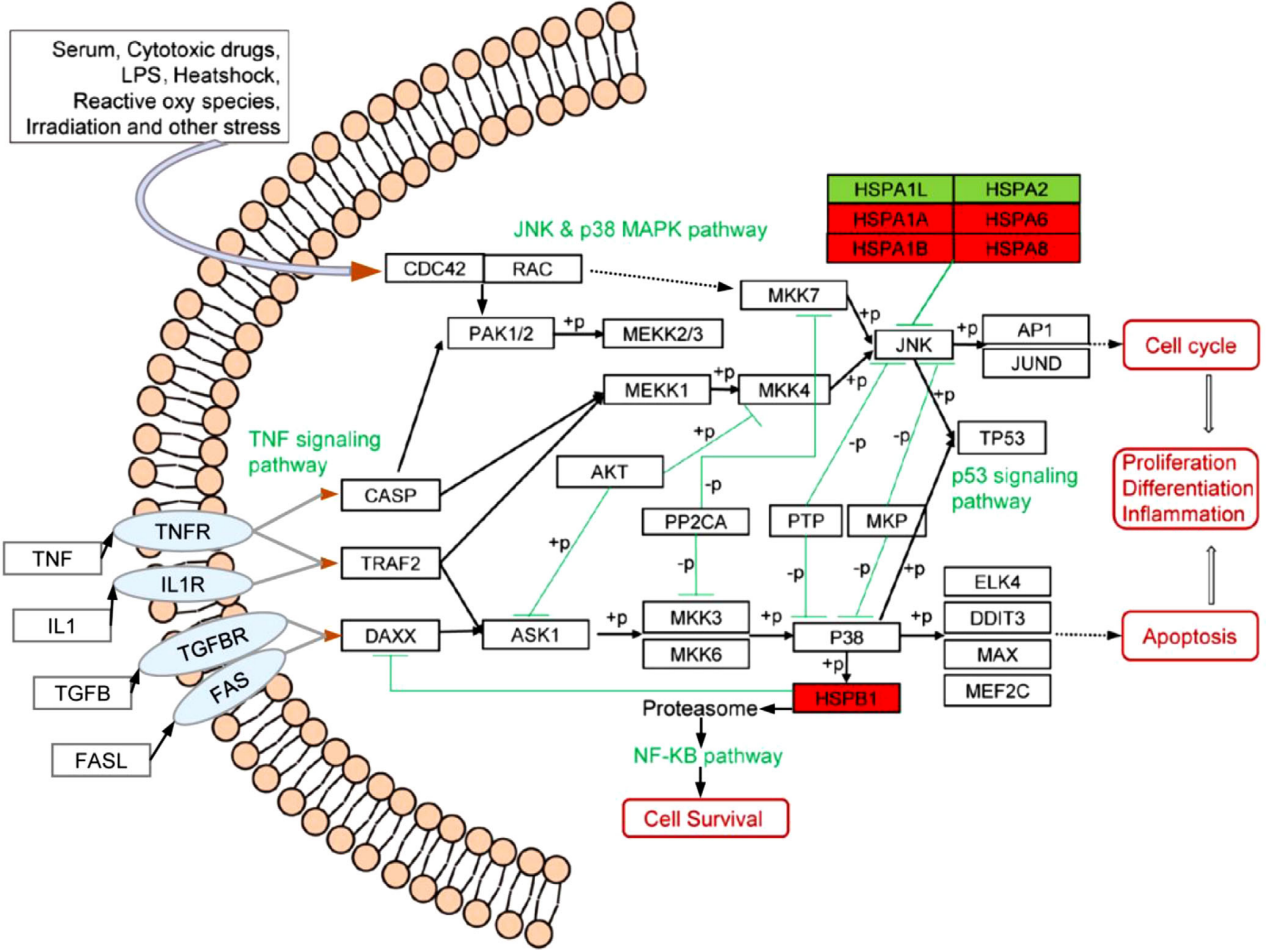
The possible mechanism of HSPs in cancer development. The red box indicates that the HSP is upregulated in most cancers; The green box indicates that the HSP is downregulated in most cancers. Green line: inhibition; “+p”: phosphorylation; “‐p”: dephosphorylation

In conclusion, expressions and mutations of HSPs family members varied among cancer types, severities, and responses to therapies. The regulatory roles of HSPs in cancer could depend upon the cancer cell origin, development, and locations, during which the interactions among HSP family members and between HSPs with other factors are critical. Our preliminary data indicate that HSPs can be a strong group of cancer‐specific biomarkers and therapy‐specific targets.

## Funding

This work was supported by the Science and technology innovation action plan of Shanghai Science and Technology Commission (Grant No.18140904002)

## Supporting information



Supporting Information
