# Supplementary material for: Variations of human heat shock proteins in multiple cancers
Source: Clin Transl Med. 2021 Feb 17;11(2):e320. doi: 10.1002/ctm2.320 (PMC7888541; doi:10.1002/ctm2.320)
Supplement: Supplementary file 1 — Supporting Information [file CTM2-11-e320-s001.docx]

Supplemental Table 1: The number of differentially expressed genes (DEG) of dysregulated (sum of upregulated and downregulated), upregulated, or downregulated heat shock proteins (HSPs) in pan-cancer with *p* values less than 0.05 and changed folds >1.5, as compared with levels of normal tissues, respectively.

Supplemental Table 2: HSP gene mutation frequencies in 28 TCGA Pan-cancer samples from cBioPortal database.

Supplemental Table 3: Receiver operating characteristic (ROC) curves of differentially expressed HSP genes in patients with lung adenocarcinoma.
